# Applications of de Bruijn graphs in microbiome research

**DOI:** 10.1002/imt2.4

**Published:** 2022-03-01

**Authors:** Keith Dufault‐Thompson, Xiaofang Jiang

**Affiliations:** ^1^ Intramural Research Program National Library of Medicine, National Institutes of Health Bethesda Maryland USA

**Keywords:** de Bruijn graphs, microbiome, Omics

## Abstract

High‐throughput sequencing has become an increasingly central component of microbiome research. The development of de Bruijn graph‐based methods for assembling high‐throughput sequencing data has been an important part of the broader adoption of sequencing as part of biological studies. Recent advances in the construction and representation of de Bruijn graphs have led to new approaches that utilize the de Bruijn graph data structure to aid in different biological analyses. One type of application of these methods has been in alternative approaches to the assembly of sequencing data like gene‐targeted assembly, where only gene sequences are assembled out of larger metagenomes, and differential assembly, where sequences that are differentially present between two samples are assembled. de Bruijn graphs have also been applied for comparative genomics where they can be used to represent large sets of multiple genomes or metagenomes where structural features in the graphs can be used to identify variants, indels, and homologous regions in sequences. These de Bruijn graph‐based representations of sequencing data have even begun to be applied to whole sequencing databases for large‐scale searches and experiment discovery. de Bruijn graphs have played a central role in how high‐throughput sequencing data is worked with, and the rapid development of new tools that rely on these data structures suggests that they will continue to play an important role in biology in the future.

## Highlights

- de Bruijn graph‐based sequence assembly approaches have been an essential part of the broad application of sequencing methods, especially in microbiome research.
- de Bruijn graphs can be used to efficiently represent sequencing data in a format that is highly scalable and can be extended and modified to address different research questions.
- de Bruijn graph‐based analysis methods have been developed for comparative genomics, the identification of genetic variants, and for large‐scale searching of unassembled sequencing data.
- The de Bruijn graph data structure will continue to be a central component of sequence assembly and analysis approaches in the future.

## Graphical abstract

The ability to efficiently assemble high‐throughput sequencing data using de Bruijn graph‐based assembly methods has been an important factor in the adoption of sequencing as a central component of microbiome research. Recent methods have applied the de Bruijn graph data structure as a component of analytical tools as well, opening up new routes of analysis in comparative genomics and metagenomics. de Bruijn graphs will likely continue to have a prominent role in how microbiome sequencing data is assembled and analyzed.

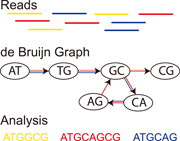

## INTRODUCTION

The rapid development and improvement of genome sequencing technology has led to significant advances in microbiome research, including the increased availability of reference genomes and the ability to sequence entire microbial communities using high‐throughput sequencing. With these technological advances have come a variety of new challenges related to how these data, often in the form of short‐read sequences, are managed, processed, and analyzed, which have been addressed through the development of new algorithms and software [1]. Some of the most significant advances in how short‐read sequencing data is handled have come from the application of de Bruijn graphs (DBGs), which are networks that represent the overlapping relationships between sequence fragments, called *k*‐mers, derived from a set of input sequences [2]. DBGs have been prominently used in genome assembly, where they have comprised a central component of many of the most efficient de novo genome and metagenome assembly algorithms [2]. Over the past decade, DBGs have also seen wider use as components of analytical tools, being applied for a wide range of tasks, including bacterial pangenome analysis, the identification of genome variants, and the comparison of Omics samples. While these methods have not been widely adopted as parts of many microbiome studies, they have shown promising results. DBGs have been instrumental in working with short‐read sequencing data and will likely continue to have significant roles as sequencing becomes an increasingly central component of studying microbes.

## APPLICATIONS OF DBGs IN GENOME AND METAGENOME ASSEMBLY

### Assembly of short‐read sequences

The problem of assembling short‐read sequences into larger genome sequences is fundamental to the use of next‐generation sequencing in microbiome research. This problem has been addressed through multiple approaches, including those employed by Greedy Assemblers [3, 4] and Overlap‐layout‐consensus assemblers [5], which rely on the identification of overlapping regions between the raw reads and reference‐based assemblers which utilize read mapping to an already assembled reference genome [6]. These methods were widely used to generate early genome assemblies and continue to be used today, but they have limitations. Both Greedy and Overlap‐layout‐consensus assembly use information about overlapping regions between reads, which can be computationally intensive to calculate, and often have problems assembling low‐complexity sequences like repeats and dealing with samples that have high sequencing depth [3]. Reference‐based assembly can produce high‐quality genome assemblies, but this method requires a genome of a closely related organism limiting its application to novel organisms and can have problems with resolving ambiguous read mapping to the reference sequence [3]. The most significant advances in short‐read assembly for genomes and metagenomes have come through the use of DBGs, which overcome many of the limitations of other assembly approaches [2, 7]. DBG‐based assembly approaches do not rely on calculating the overlap between reads, avoiding this computationally intensive step involved in greedy and overlap‐layout‐consensus assembly, and they only require the sequencing reads circumventing the need for a reference genome [2, 7]. DBG‐based assembly can be sensitive to sequencing errors, which can introduce additional noise to the graph [3], but overall the advantages of DBG‐based methods have led to the broad adoption of DBG‐based assembly for the assembly of short‐read genomic and metagenomic data.

**Figure 1:**
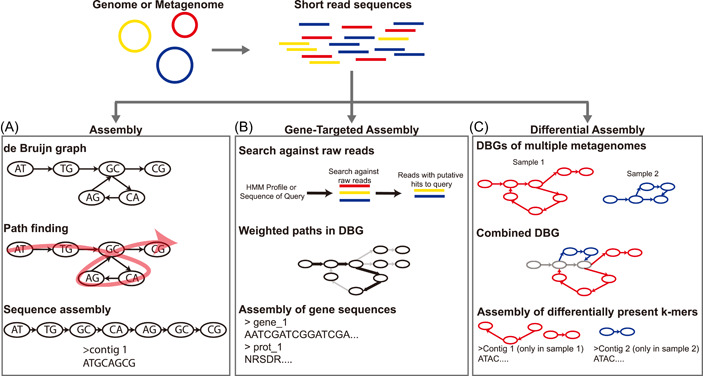
Illustration showing different applications of de Bruijn graphs in genome and metagenome assembly. (A) Illustration of de Bruijn graph assembly. First, a de Bruijn graph is constructed from raw reads, then a path through the graph that visits each *k*‐mer is identified (red arrow over the graph), and lastly a sequence is assembled based on this path. (B) Illustration of the general process for gene‐targeted assembly. First reference sequences or profiles are used to identify reads that may contain partial gene sequences, next this information is used to add weights (thicker black arrows) to the graph, and lastly these weighted paths can be used to directly assembly gene sequences. (C) Illustration showing the concept of differential assembly. de Bruijn graphs are generated from multiple metagenomes (red and blue graphs). These de Bruijn graphs can then be combined revealing portions of the graph that are shared between the two metagenomes (gray nodes and edges), or portions that are unique to one metagenome (red or blue nodes and edges). Sequences that are uniquely present in one sample versus the other can then be assembled

DBG‐based genome assembly starts with decomposing the raw sequencing reads into subsequences of *k* length called *k*‐mers. A graph is then constructed by first defining a prefix, a *k*‐mer minus the last nucleotide, and a suffix, a *k*‐mer minus the first nucleotide, for every *k*‐mer. The total set of unique suffixes and prefixes form the nodes in the graph and the edges are added based on the *k*‐mers that link a given suffix and prefix. The assembly of longer sequences is then done by finding an Eulerian cycle in the graph, a path that visits each edge (representing a *k*‐mer) in the graph one time, and then collapsing the sequence of the *k*‐mers in this path to assemble longer sequences [2] (Figure 1A). DBG‐based genome assembly does not require the calculation of alignments between reads, and has allowed for the efficient and scalable assembly of sequencing data [2]. Early DBG‐based assemblers, including EULER [8], EULER‐SR [9], Velvet [10, 11], and ALLPATHS [12, 13], employed the basic strategy described above with modifications to address specific challenges like how repetitive sequences are assembled and how sequencing errors are detected and handled. Later assembly approaches, like those employed by the SPAdes family of software [7, 14], SOAPdenovo family of software [15, 16], and MEGAHIT [17], built upon many of the concepts employed by the early assemblers, with a focus on improving efficiency, handling larger datasets like those from metagenomes, and improving the accuracy of the assemblies. Overall, these DBG‐based assembly tools represented a significant step forward in sequence assembly, overcoming many of the challenges that hindered older assembly approaches and leading to their wide use in microbiome studies for the assembly of sequence data.

### Gene‐targeted assembly

In many microbiome surveys, one of the desired outcomes is the identification of genes of interest that could be used as phylogenetic markers, signals of disease, or represent unique functions. While metagenome assembly has improved dramatically, some challenges remain, including bias toward dominant members of the microbial community, leading to rarer genes being missed and metagenome assembly can have significant computational costs [18]. Gene‐targeted assembly approaches seek to address these challenges by assembling gene sequences directly from metagenomes rather than predicting them from assembled contigs. Many gene‐targeted metagenome assembly approaches utilize DBGs during the assembly process. These approaches typically use either a sequence or profile hidden Markov model‐based search against the raw reads to identify reads that likely contain portions of gene coding sequences. Some methods, like those applied by Xander [19] and MegaGTA [20], then use this search information to modify the de Bruijn assembly graph by adding weights to specific paths in the graph (Figure 1B), aiding in the identification and assembly of gene sequences. Other tools, including SAT‐Assembler [21], MEGAN‐Assembler [22], and phyloFlash [23], use the search results to filter the raw reads so that only reads with likely coding sequences are used during the assembly process. Some of these gene‐targeted assembly approaches have utilized extended versions of DBGs, highlighting the flexibility of DBGs in different kinds of analyses (Table 1). These modified DBGs include the weighted DBG graphs used by Xander [19] and MegaGTA [20], amino acid‐based DBGs like those used in MetaPA [24], and a variation of a DBG called a succinct DBG (sDBG) employed in MegaGTA [20]. sDBGs are memory efficiency variations of DBGs designed to be applied to large datasets like those generated from metagenomes and bacterial pangenomes [25] and have been adopted by multiple DBG‐based assembly and analysis methods, including MegaGTA [20], MetaGraph [26], and MEGAHIT [17]. Gene‐targeted assembly can facilitate the analysis of metagenomes data while avoiding some of the potential biases associated with the assembly process. This allows for the identification of genes from rarer species in the community and can provide a more complete view of what organisms and genes are present in a community based on metagenome sequencing.

**Table 1:** Common modifications applied to the basic de Bruijn graph (DBG) data structure and examples of applications that utilize them

Modification	Key concept	Applications
Coloring	Each *k*‐mer in the DBG is associated with annotation information describing its original source (e.g., genome, read)	MetaGraph, TwoPaCo, Cuttlefish, Bifrost, Cortex, MCCortext, DiscoSnp, Bubbleparse, Scalpel, LUEVARI, Rainbowfish, Mantis, VARI
Succinct representation	Data in the DBG is represented as a bit vector or other space‐efficient representation	MegaGTA, MEGAHIT, MetaGraph, Rainbowfish
Simplification/compaction	*K*‐mers in the graph are collapsed into larger linear sequences and bubbles or tips caused by potential errors are removed from the graph	Simpletigs, splitMEM, MetaGraph
Weighting	Additional data is used to add weights to paths in the graph which can be used in subsequent assembly and analysis	Xander, MegaGTA

### Identification of microbial species from metagenomes

One of the common goals of microbiome research is to identify what bacteria are present and what genes they have. This information can be obtained using metagenomics, but this requires the ability to differentiate which reads and contigs come from different species so that the potential roles of the organisms can be better understood. The utility of the DBG in identifying different microbial strains in metagenomes was demonstrated by Wang et al. [27], where they utilized read mapping to a metagenome assembly DBG to differentiate reads derived from different bacterial strains without the use of reference genomes. Many recent efforts have been focused on deriving nearly complete microbial genomes from metagenomic reads. These metagenome‐assembled genomes (MAGs) are generated by binning assembled contigs based on nucleotide frequency and read coverage, relying on the assumption that these factors will differ between the species in the original community [28, 29]. Recent attempts at improving metagenomic binning have incorporated the DBG to help make and refine MAGs. These methods, including GraphBin [30] and METAMVGL [31], incorporate structural features of the DBG, like the connections between *k*‐mers and the presence of unconnected components of the graph, to refine which contigs are included in each MAG. These approaches highlight the utility of DBGs in downstream analyses, where information already present in the DBG can be used to improve subsequent analyses and may greatly improve the recovery of higher quality MAGs.

### Comparison of Omics samples and differential assembly

As metagenomic sequencing becomes less expensive, it is becoming a more commonly applied approach, and studies will often involve sequencing multiple metagenomes. This has led to the need for efficient ways to identify similarities and differences between metagenomes derived from different samples. Still, the size and complexity of these data makes this a difficult challenge. Recent studies have proposed DBG‐based approaches for making these comparisons. EMDeBruijn utilizes DBGs generated from multiple microbiomes and applies a statistical approach to compare the distances between different samples. This approach has been used to look at viral populations and aid in the characterization of hepatitis C transmission, demonstrating its utility in different kinds of biological analyses [32]. Similarly, MetaFast uses a simplified DBG constructed from multiple metagenomes to quantify their similarities, providing a way to compare diversity between different environments or samples [33]. The recently proposed MetaGraph approach shows significant promise, allowing for the indexing and querying of entire sequencing databases or multiple metagenomes in an efficient DBG‐based format [26]. One of the widely applicable uses of a method like this would be in what the authors call “differential assembly,” where the MetaGraph DBG can be used to identify *k*‐mers found in some metagenomes but not others which can then be assembled and analyzed to look at differences in the microbial communities between samples [26] (Figure 1C). These methods for comparing metagenomes without the need for costly read mapping between samples or the use of a reference database have broad applications and make the efficient and accurate comparisons of Omics samples possible.

## COMPARATIVE GENOMICS AND METAGENOMICS USING DBGs

### Comparative genomics using colored DBGs

**Figure 2:**
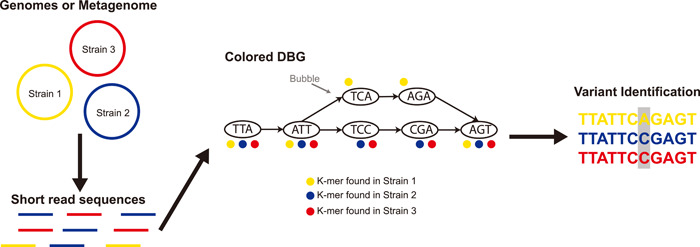
Illustration showing the concept of a colored de Bruijn graph (DBG) and the process of variant identification using the graph

The identification of genetic variants between microbes, like single‐nucleotide variants (SNVs) and indels, has broad applications in biomedical and ecological studies [34], monitoring outbreaks of pathogens [35, 36], and differentiating microbial populations at the strain level [37]. Many of the standard approaches used for variant discovery utilize mapping to a reference genome or sequence, which can be computationally costly and may not always be possible when references are not available or are too divergent to be used for accurate comparisons. To address this problem, multiple tools have been developed for reference‐free variant detection using DBGs. These approaches typically utilize a variant of the DBG called a colored de Bruijn graph (cDBG), which is a DBG constructed from multiple sources, for example, multiple genomes or different metagenomic samples, where the *k*‐mers are assigned different “color” annotations based on which inputs they were present in [38] (Figure 2, Table 1). Multiple tools have been created to facilitate the construction of these cDBGs from either collections of genomes or multiple sets of raw reads, including TwoPaCo [39], Bifrost [40], and Cuttlefish [41]. The ability to construct these dBGS has facilitated the growth of multiple other tools that have focused on identifying genetic variants using DBGs without the need for a reference genome.

Most of these tools have been developed for the identification of genetic variants either based on a set of assembled genomes or based on raw reads from sequencing different individuals of the same species. For detecting SNVs, tools like Cortex [38, 42], MCCortex [43]. DiscoSnp [44], and Bubbleparse [45], have all been developed based on the analysis of DBGs or cDBGs to identify structural features often referred to as “bubbles” in the graph, which are points where parallel paths formed by different *k*‐mers diverge and then converge back (Figure 2), which may contain SNVs. These concepts have been subsequently expanded to facilitate the detection of more complex genetic variants like small insertions and deletions in the tools DiscoSnp++ [46] and Scalpel [47]. This process has been extended in the BubbZ approach to utilize a compacted representation of the DBGs to detect homologous regions between genomes allowing for comparative analyses between different genomes without the need for whole‐genome alignments [48] (Table 1). These approaches have many potential applications in microbiome research, where high‐quality reference genomes for many microbial strains are often lacking, and reference‐free approaches would open multiple new routes for analyzing microbial communities and isolates.

### Reference‐free single nucleotide variant calling in metagenomes

Identifying genetic variants in metagenomic samples is significantly more challenging than it is when dealing with sets of genomes. Metagenomic samples can have many microbial species present, contain multiple closely related strains, and different organisms may have closely homologous genes, all of which would make applying traditional variant calling approaches in metagenomes difficult. Many of the same techniques previously described for variant identification can be used or adapted for the analysis of metagenomic datasets, including Cortex [38, 42], DiscoSnp [44], DiscoSnp++ [46], and Scalpel [47] with the same concepts being applicable to metagenomic DBGs. While these methods can be applied to metagenomic data, not many tools have been developed specifically for the task of reference‐free variant identification from metagenomes. The recently published LUEVARI approach utilizes a cDBG where the coloring of the graph is based on the reads in the metagenome leading to more significantly more sensitive variant identification from metagenomes compared to other tools [49]. These approaches to variant identification could have major implications for microbiome research, where metagenomic sequencing is quickly becoming a standard approach for investigating the microbiome.

### Querying Omics data sets and experiment discovery

With the scale and amount of Omics data being produced, the need has arisen for efficient methods to query these data. Performing searches on already assembled datasets has drawbacks, including being limited by the efficiency of the search approach, having different quality assemblies made using different approaches, and being limited to the small subset of data that is available as assembled data [50]. Multiple methods have been developed recently to facilitate the construction of cDBGs from large datasets, including the entire databases like the Sequence Read Archive (SRA) from NCBI, and the subsequent development of search methods that can be used to query these cDBGs has allowed for their use in large scale searching and experiment discovery. A vital component of these advances has been the development of compact versions of colored DBGs like the sDBG [25], Rainbowfish DBG [51], Cuttlefish DBG [41], splitMEM [52], and the Simpletigs DBG [53], which employ various methods to reduce the size of, complexity of, and memory needed to store the DBG and coloring data (Table 1). These more efficient representations of DBGs are highly scalable, meaning they can be efficiently applied to extremely large data sets, and multiple approaches for performing searches on these graphs have been developed. The Mantis and VARI programs utilize an index based querying approach to identify which *k*‐mers from a given query are present in different sequence datasets [54, 55] and was able to efficiently query for the presence of all known human transcripts in the SRA database [55] and to query metagenomic samples from food production facilities [54]. Similarly, the recently proposed MetaGraph includes a *k*‐mer matching‐based search and a sequence‐to‐graph alignment‐based search approach for querying their MetaGraph indices [26]. One of the major challenges facing microbiome research is experiment discovery, or how to identify sequencing projects that contain a gene of interest among the rapidly growing databases of sequences [56]. These DBG‐based approaches not only allow for these large sequence databases to be represented as concise cDBGs, but also allow for efficient searching of these indexed datasets allowing for their broader application in microbiome research.

## APPLICATIONS OF DBGs IN TRANSCRIPTOMICS AND PROTEOMIC

DBGs have also been used to analyze transcriptomic and proteomic data. These other types of Omics data bring their own unique challenges and the methods used to analyze them differ from the approaches applied to metagenomes [57, 58]. The assembly and analysis of these types of Omics data often rely on reference databases, but they often fail to capture underrepresented or novel transcripts and proteins [59]. The approaches that utilize DBGs have sought to overcome this issue by using paired Omics data, where a DBG constructed from a metagenome sequenced from the same sample is used to aid in the assembly and analysis of a metatranscriptome or metagenome [59]. Read2Graph relies on the alignment of reads from a metatranscriptome to a DBG generated from a paired metagenome, resulting in a significant improvement in the assembly of transcripts compared to de novo metatranscriptome assembly approaches [59]. Similarly, the Graph2Pep and Graph2Pro approaches use a paired metagenome or metatranscriptome to greatly improve the identification of peptides in a metaproteomic sample [60]. In addition to assembly, read mapping to DBGs has been applied to help with the identification of splicing and to perform more accurate expression estimates from RNA‐seq data [61]. The efficient assembly and analysis of metatranscriptomic and metaproteomic data have been a major challenge, limiting the broader application of these approaches in different studies. The development of these efficient graph‐based analysis approaches has major potential and can allow for the broader application of multiomics approaches in increasingly complex biological systems.

## THE FUTURE ROLE OF DBGs IN MICROBIOME RESEARCH

The study of microbial communities through high throughput sequences has become an integral component of biomedical and environmental microbiology. The continued development of methods to efficiently assemble and analyze sequencing data has been instrumental in the broad adoption of sequencing in biological studies, and DBGs specifically have been a central component of many of these methods. DBGs have been an essential component of short‐read assembly methods and approaches for the assembly and analysis of long‐read sequencing data are already being developed, demonstrating their application to this rapidly growing technology [62]. Additionally, significant algorithmic advances dealing with the efficient construction [39, 41] and representation of DBGs [50, 53, 63] continue to be made which will provide a foundation for the development of new methods. While DBGs will undoubtedly continue to play a central role in assembly, their use in analytical tools has also been rapidly increasing over the past decade. These DBG‐based methods have proved to be efficient and highly scalable, allowing for their application to extremely large datasets and opening new routes of biological discovery that can leverage the ever‐increasing amount of available Omics data. As sequencing becomes less expensive and even more widely applied, DBGs will continue to be at the center of many tools used across microbiome research.

## CONFLICT OF INTERESTS

The authors declare that there are no conflict of interests.

## AUTHOR CONTRIBUTIONS

Keith Dufault‐Thompson and Xiaofang Jiang wrote the manuscript.
